# Reducing the Climate Impact of Critical Care

**DOI:** 10.1016/j.chstcc.2023.100037

**Published:** 2024-03

**Authors:** Alexander S. Rabin, Peggy S. Lai, Stephanie I. Maximous, Hari M. Shankar

**Affiliations:** Division of Pulmonary and Critical Care, Department of Medicine, University of Michigan, Ann Arbor, MI; Pulmonary Section, Veterans Affairs Ann Arbor Healthcare System, Ann Arbor, MI; Division of Pulmonary and Critical Care, Department of Medicine, Massachusetts General Hospital, Harvard Medical School, Boston, MA; Division of Pulmonary, Allergy, Critical Care, and Sleep Medicine, Department of Medicine, University of Pittsburgh, Pittsburgh; Division of Pulmonary, Allergy and Critical Care, Department of Medicine, University of Pennsylvania, Philadelphia, PA

**Keywords:** advocacy, climate change, critical care, decarbonization, health care sustainability, waste

## Abstract

As the health effects of climate change intensify, critical care providers have an urgent responsibility to minimize the environmental impact of health care delivery. Although the response of critical care clinicians in managing climate-exacerbated diseases such as asthma and heat stroke is well recognized, the impact of critical care delivery on climate change itself may be less familiar. This case-based review explores the drivers of the ICU climate footprint, including high energy and electricity use, supply chain contributions, pharmaceutical greenhouse gas emissions, plastic waste, and low-value care. Potential solutions then are presented for each of these elements, with an emphasis on multidisciplinary team engagement to enact lasting change. The role of the ICU clinician as environmental policy advocate also is explored. Despite the grave clinical implications of the climate crisis, critical care providers are well positioned to mitigate their own climate impacts and to help lead health care decarbonization.

The health impacts of climate change are pervasive and deadly. Heat waves, hurricanes, vector-borne diseases, wildfires, and malnutrition all are on the rise as the planet warms.^1,2^ At the center of it all are critical care providers, who serve the most vulnerable patients in their time of greatest need. Studies have shown how the effects of climate change on critical illness are not just theoretical: heat waves have been associated with increased hospitalizations among older adults,^3^ whereas the proliferation of infectious diseases like Dengue places new demands on pediatric ICU providers.^4,5^ Individuals from racial and ethnic minority groups who have experienced historic health inequities are among those in serious jeopardy.^6,7^ According to one Australian analysis, projections of critical care needs with unrestrained greenhouse gas (GHG) emissions suggest a near doubling in that country’s respiratory-related ICU admissions by 2090.^8^

It is ironic, then, that the hospital services most affected by the climate crisis bear significant responsibility for exacerbating the problem. Intensive care produces more than triple the GHG emissions per day compared with other inpatient care: 138 kg vs 45 kg CO_2_ equivalent.^9^ Just 5 days of ICU care for sepsis has been shown to produce the emissions equivalent of a 10-h flight.^10^ High energy demand for heating, ventilation, and air conditioning (HVAC); the pervasive use of disposable products; a high reliance on pharmacologic agents; and the costly and widespread use of diagnostic tests all contribute to this heavy environmental toll (Table 1).

Although ICU-related GHG emissions may seem trivial in the larger context of the global climate crisis, they add up. The health sector as a whole accounts for approximately 5% of global GHG emissions.^11^ If health care were a country, it would be the fifth largest carbon emitter, ahead of the entire country of Japan.^2^ Leading the pack is the US health sector, whose climate emissions comprise one-quarter of global health care GHG emissions and have risen a further 6% from 2010 to 2018.^2,12^

In contrast, the United Kingdom’s National Health Service has been able to reduce its GHG emissions—by 26% overall and by 64% per inpatient admission—despite doubling the number of inpatient hospital encounters between 1990 and 2019.^13^ Variability in the climate footprint within countries and between medical practices^14^ suggests that changes at the institutional or individual provider level may drive down health-related GHG emissions effectively.

Despite growing concern about the climate footprint of the health care system, less attention has been paid to emissions stemming from the ICU. In this case-based discussion, we explore the climate impact of ICU care and identify areas for improvement. We also underscore the importance of physician advocacy to promote broader societal changes to help address the crisis.

## Case Presentation

A 79-year-old woman with a medical history of severe COPD and dementia sought treatment with 2 days of dyspnea on exertion, cough, and sputum production that began during a wildfire smoke event. She reported extreme breathlessness and difficulty walking around her apartment. More frequent use of her only respiratory medication, an albuterol metered-dose inhaler (MDI) administered up to 12 puffs/d, provided minimal relief.

In the ED, the patient was found to be in respiratory distress with increased work of breathing and a weak cough. Arterial blood gas analysis demonstrated respiratory acidosis with normal oxygenation. A respiratory viral panel showed negative results for all common viral pathogens. The chest radiograph demonstrated hyperinflation without consolidation. IV corticosteroids, MDI bronchodilators, and macrolide antibiotics were administered. She was administered bilevel noninvasive positive pressure ventilation, but subsequently vomited into the mask. A decision was made to perform endotracheal intubation, and she was admitted to the ICU in a respiratory isolation room. After stabilization, the patient underwent CT scan pulmonary angiography showing severe emphysema without evidence of pulmonary embolism.

On hospital day 6, the patient demonstrated a fever. Daily laboratory studies were notable for a worsening leukocytosis, whereas the daily chest radiograph showed development of a new left lower lobe lung opacity. Empirical vancomycin and cefepime were initiated. A single-use fiberoptic bronchoscope was used to obtain a BAL specimen for culture analysis. The bronchoscopy was repeated on hospital days 8 and 9 for clearance of secretions.

The patient’s hospital course was characterized by recurrent sepsis, acute renal failure resulting in initiation of renal replacement therapy, and a prolonged cardiac arrest resulting in anoxic brain injury. Attempts to liberate her from the ventilator were limited by secretions, delirium, and neuromuscular weakness. A tracheostomy and gastrostomy tube were placed, and on hospital day 36, the patient was transferred to a long-term care facility.

### Energy and Electricity Use

Hospital energy generation, distribution, and use account for approximately 40% of US health care sector GHG emissions.^2^ At least three-quarters of ICU GHG emissions originate from HVAC systems; ICU equipment, patient monitors, and computers account for only 10% of electricity use.^10^ HVAC systems are energy-intensive machines that enable a comfortable and safe work environment by treating and filtering air. The ICU, which requires frequent air exchanges to prevent the potential spread of infection among critically ill or immunocompromised patients, pushes energy use even higher. Notably, the air is treated and exchanged regardless of whether a patient occupies the bed.

Although most HVAC systems derive their energy from fossil fuels, hospital emissions vary widely across geographic settings according to the predominant regional energy source (eg, coal, renewables, nuclear).^15^ The climate footprint of a given ICU thus reflects the electricity mix of the local or national grid.^16^ This wide range of ICU-related emissions has been illustrated in a study comparing patients undergoing treatment for septic shock in a US and an Australian ICU in which the CO_2_-equivalent emissions ranged from 88 kg/patient/d in an Australian ICU to 178 kg/patient/d in the US ICU.^10^

## How We Do It

The provenance of the hospital’s energy may not be foremost in the minds of most ICU clinicians, but we believe it should be. The position of respect and leadership that health care providers command can be translated beyond the bedside to energy efficiency campaigns and even electricity purchasing agreements. Although the path toward reducing ICU energy use differs based on setting, we suggest the following: (1) harnessing opportunities to close or limit airflow to unoccupied ICU rooms to minimize wasted HVAC use and decreasing off-hour equipment energy use through mechanisms such as automatic light dimming and computer sleep modes; (2) partnering with leaders in the hospital’s engineering, physical plant, or utilities departments to decrease ICU energy consumption; and (3) engaging hospital leadership to transition away from fossil fuel toward fully renewable energy sources, including through the use of renewable energy power purchase agreements.

### Equipment Procurement, Plastics, and Waste

The care of a critically ill patient requires a massive volume of medical supplies such as procedure kits, pharmaceuticals, tubing, linens, bedpans, and personal protective equipment (PPE). Indeed, the vast international supply chains required to supply the ICU constitute the bulk of its climate footprint. These so-called scope 3 emissions—generated from the harvesting of raw materials, manufacturing, transportation, and ultimately waste disposal—account for 60% to 82% of health care’s total GHG emissions.^12,17^

The problem has only worsened over the last few decades as the US health care industry increasingly relies on single-use disposable items, generating approximately 14,000 tons of medical waste daily, of which 20% to 25% is fossil fuel-derived plastics.^18^ These single-use products consist not only of common items such as gloves and other PPE, but now also include traditionally reusable (and often sophisticated) devices such as BP cuffs, pulse oximeters, bronchoscopes, and laryngoscopes.

Research echoes what many clinicians have long known through experience: that the amount of solid waste produced in critical care settings is significantly more than that produced in other acute care units. One study conducted in a US hospital suggested that the ICU produced almost 30% more solid waste per bed per day compared with a comparable non-ICU inpatient bed in the same facility.^9^ The COVID-19 pandemic has only amplified these trends, leading to large increases in the amount of waste owing to the widespread, and sometimes needless, use of PPE.^19^ This waste then must be transported to landfills or incinerators, where it may pollute local waterways, soil, and air,^20^ often in communities of color or of low socioeconomic status. In this way, health care pollution has been externalized further to vulnerable communities both domestically and internationally. Meanwhile, attempts to increase equipment reprocessing and reuse may be constrained by limits on local water and energy resources that could impede the efficiency of reprocessing efforts.

### How We Do It

Critical care clinicians have expertise and direct influence over the tools that are used in the ICU, both as individuals and by virtue of the hospital’s purchasing power. Equipment procurement can and should be within the clinician’s purview because procurement decisions have an outsize influence over the ICU’s climate footprint and health care outcomes.

Recommendations include: (1) prioritizing reusable equipment over single-use products, acknowledging that the reduction in climate impact with reuse or recycling may vary by hospital location, energy use, water resources, and other factors^21–23^; (2) advocating for the reprocessing of tools and devices by the hospital or supplier as well as modernization of infection prevention practices and related regulatory reforms^24^; (3) encouraging bedside practices that result in less waste (eg, minimizing stock of disposable products inside patient rooms to limit disposal of unused supplies and re-evaluating protocols governing frequency of routine changes of IV infusion sets^25^); (4) strengthening evidence-based infection control practices to avoid overuse of single-use products including PPE^24,26^; and (5) increasing ICU clinician representation on hospital procurement and supply chain committees to incorporate environmental benchmarks better.

### Pharmaceuticals

Pharmaceutical products form the backbone of therapeutic intervention in the ICU, yet contribute disproportionately to health care’s climate footprint. Although the critical care practitioner may feel disconnected from their hospital’s energy procurement or supply chain, the same clinician wields substantial influence over medication choices such as the choice of inhaler device or anesthetic gas. Folding environmental considerations into prescribing practice thus can yield environmental benefits without compromising the quality of care.

MDIs—devices essential to the management of respiratory disease exacerbations in the ICU—present one area of particular concern. MDIs contain hydrofluorocarbon propellants that trap heat in the atmosphere more than 1,000-fold more strongly than CO_2_.^27^ The use of one albuterol inhaler has a similar global warming effect to driving a passenger car up to 200 km.^28^ Estimates are lacking in the United States, but hydrofluorocarbon gases comprise an estimated 3% to 4% of the UK National Health Service GHG emissions.^29^ Compounding the problem, inhalers often are wasted,^30^ left unused on a bedside table, or tossed in the garbage after the patient transfers out of the ICU. Even when the devices are administered on schedule, little consideration may be given toward finding an inhaler device that best matches the patient’s needs or abilities.^31^

Fortunately, strong precedent exists for improving climate-conscious prescribing: for example, many operating rooms are beginning to limit the use of fluorinated anesthetic gases. Several health systems have adopted measures to minimize use of anesthetics like desflurane and nitrous oxide that are potent GHGs. One provider-led educational initiative coupled with an intervention to limit the use of desflurane, a potent GHG with global warming potential 40 to 50 times that of similar agents,^32^ resulted in meaningful climate benefits.^33^ Major health systems now have adopted measures to reduce or eliminate the use of desflurane and other anesthetic gasses with high heat-trapping effect,^34,35^ to limit the gas flow of inhaled anesthetics, and to consider a transition to total IV anesthesia.^36^ Prolonged use of inhaled anesthetics as ICU sedatives also should be of concern; this approach recently was subject to a surge of interest after the IV drug shortages that accompanied the COVID-19 pandemic.^37^

### How We Do It

In many instances, excellent therapeutic alternatives are available to pharmaceuticals with high global warming potential, including hydrofluorocarbon-containing inhalers. However, even when MDIs are required, a more thoughtful approach, such as provision of a spacer device^38^ in tandem with device education, may have lasting health benefits beyond the ICU. Other strategies include: (1) focusing on sound disease diagnosis and longitudinal chronic disease management that may reduce the need for rescue MDI use and emergency or critical care use; (2) administering inhaled medications as nonpropellant-based dry-powder or soft-mist inhalers, when deemed clinically equivalent, for long-term disease control; (3) incorporating climate footprint into medication purchasing decisions, formularies, and electronic health record ordering menus^39^; and (4) encouraging research on pharmaceutical life-cycle assessments to understand better the net environmental benefits or harms of clinical products such as MDIs.^40^

### Low-Value Care

Inappropriate ICU care leads to worse patient outcomes, moral distress, increased health care costs, and environmental degradation. Low-value care—defined as overtreatment, failure of care coordination and execution, pricing failure, and abuse—accounts for up to 20% of total health care.^41^ This statistic likely is even higher in the ICU, where futile care, which provides minimal long-term benefit to the patient yet results in high resource use, is common.^42^ Although direct estimates are lacking, the damage inflicted on the patient and the climate from unnecessary ICU interventions likely is substantial.

### How We Do It

Minimizing low-value care can have dual benefits: improving the quality of clinical care and lowering GHG emissions.^43^ Solutions may include: (1) arranging family meetings to identify goals of care soon after admission to the ICU; (2) involving palliative care consultative services and establishing goals of care early in the hospital course to avert potentially unnecessary ICU transfer if identified as futile or not consistent with patient values^44^; (3) minimizing wasteful interventions (eg, limiting routine daily phlebotomy or chest radiography and adhering to evidence-based blood transfusion thresholds)^45^; and (4) ensuring adherence to society guidelines and evidence-based therapies to minimize inappropriate treatment and low-value care.

### Intensivists as Advocates

Providers’ first-hand experiences and patient care stories lend extra legitimacy and power to climate advocacy efforts in the hospital and beyond. Importantly, advocacy takes many forms and does not always require special expertise. Hospital sustainability, or green teams, for instance, typically include experts in medical engineering, food services, and procurement, yet these committees also benefit from clinicians whose voices provide insight into the realities of patient care. Recognition and support for hospital sustainability efforts are increasing: the recently announced Sustainable Healthcare Certification program from The Joint Commission offers tools and incentives for facilities and clinicians seeking to reduce GHG emissions.^46^

Outside the hospital, clinicians can engage with local organizations working to combat climate change, can participate in direct legislative advocacy, or can promote sustainable practices within their medical societies. Speaking and writing about health care sustainability also are powerful tools for change, from the bedside to the halls of Congress.

### How We Do It

Critical care practitioners may advocate for health care sustainability in many ways, including: (1) joining a hospital sustainability team; (2) petitioning hospital leadership to join the US Department of Health and Human Services’ Health Sector Climate Pledge, which aims to reduce health system emissions by 50% by 2030 and achieve net-zero emissions by 2050; (3) encouraging national professional societies such as CHEST and the American Thoracic Society to make climate change, health, and sustainability a central pillar of research, advocacy, and education; (4) engaging with elected officials to promote climate-forward legislation; (5) publishing opinion essays in the lay press and perspective pieces in professional journals to engage wider audiences and to disseminate climate-focused education; and (6) calling for increased research, investment, and regulatory action to accelerate the development of reusable medical products, including climate-friendly inhalers.^39^

### Discussion

The case of an older woman with wildfire smoke inhalation leading to debilitating complications illustrates how the goals of quality patient care and sustainability frequently converge. From the outset, the patient’s home medication regimen, which relies on a short-acting albuterol MDI, did not follow evidence-based guidelines for the management of COPD.^47^ Maintaining respiratory disease control has been shown to improve clinical outcomes, reducing risk of exacerbation and hospitalization as well as minimizing MDI-related GHG emissions.^48^

After the patient was admitted to the hospital, her care was marked by excessive resource use. She was placed in an isolation room unnecessarily, prompting excessive use of PPE, including gowns, gloves, and masks. The chest CT scan was of dubious benefit given the low clinical suspicion for pulmonary embolism; so too, were the routine daily laboratory studies, radiographs, and bronchoscopies.

It becomes evident that the aggressive care provided is likely of low value in the context of her dementia and severe underlying pulmonary disease—and after a cardiac arrest, arguably is futile. No mention was made of palliative care consultation, which has been shown to reduce health care use at the end of life.^49^

**FIGURE 1 –:**
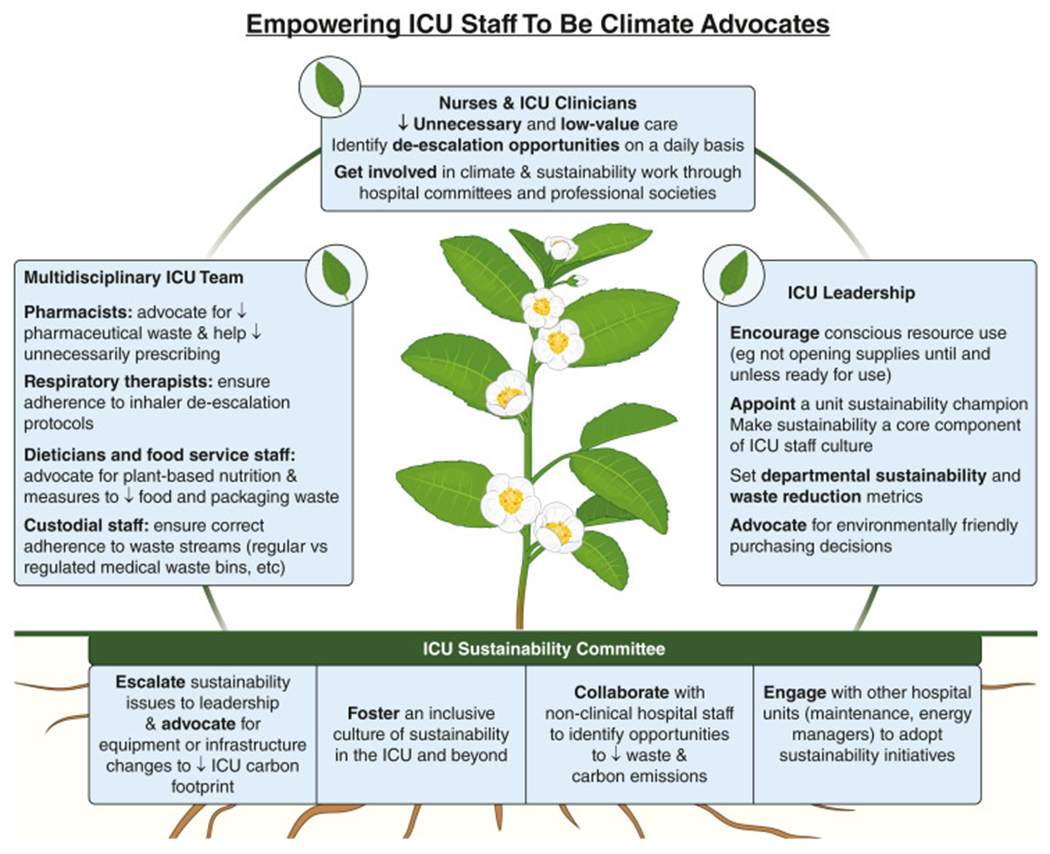
Opportunities to empower ICU staff to be climate advocates. ↓ = decrease.

The patient’s ICU course overall is characterized by an excessive contribution to health care waste without a clear clinical benefit. A multidisciplinary team of clinicians, pharmacists, nurses, respiratory therapists, and other staff can commit to establishing a culture of sustainability and can focus on waste reduction to combat these entrenched practices (Fig 1).

### Conclusions

By its very nature, the ICU is intensive. To date, this intensity has been reflected not just in patient care, but also in the ICU’s unacceptable climate toll. Climate change forces us to rethink existing systems of care and to find ways to reduce the environmental damage for which we must take responsibility.

As intensivists, we pride ourselves on our focused clinical reasoning and swift action. We should harness the same urgency, vigor, and clarity with which we attack sepsis or respiratory failure to advocate for decarbonization during this pivotal decade in the fight against climate change. Society always has relied on clinicians to respond in times of crisis, and our oath demands that we lessen the impact of our well-intentioned but harm-laden interventions. The threats posed by this crisis are irreversible and incurable, endangering patients, the next generation, and indeed all biodiversity on earth. The work of critical care medicine traditionally occurs long after missed opportunities for disease prevention; by prioritizing sustainable practices in the ICU, we can engage in population-level disease prevention and can improve the health of our communities by decreasing our climate impact immediately.

## Figures and Tables

**TABLE 1 ]:** Striking Facts About the Climate Footprint of Health Care

If the global health care sector were considered a country, it would be the fifth largest contributor to global greenhouse gas emissions.
US health care greenhouse gas emissions rose 6% from 2010 to 2018 and is the highest rate among industrialized nations.
ICU beds generate more than three times the climate footprint of regular inpatient beds.
A stay in a US ICU for septic shock has the same daily climate footprint as a one-way economy class flight for one passenger to travel from New York City to Mexico City.
The use of one albuterol inhaler has a similar global warming effect to driving a passenger car up to 200 km.

## ABBREVIATIONS:

GHG: greenhouse gas
HVAC: heating, ventilation, and air conditioning
MDI: metered-dose inhaler
PPE: personal protective equipment
